# Multi-task deep learning for glaucoma detection from color fundus images

**DOI:** 10.1038/s41598-022-16262-8

**Published:** 2022-07-20

**Authors:** Lucas Pascal, Oscar J. Perdomo, Xavier Bost, Benoit Huet, Sebastian Otálora, Maria A. Zuluaga

**Affiliations:** 1 Data Science Department, EURECOM, 06410 Sophia Antipolis, France; 2 Orkis, 13290 Aix-en-Provence, France; 3 School of Medicine and Health Sciences, Universidad del Rosario, Bogotá, Colombia; 4 Median Technologies, 06560 Valbonne, France; 5 Support Center for Advanced Neuroimaging, University Institute of Diagnostic and Interventional Neuroradiology, 3010 Bern, Switzerland

**Keywords:** Optic nerve diseases, Computer science, Biomedical engineering

## Abstract

Glaucoma is an eye condition that leads to loss of vision and blindness if not diagnosed in time. Diagnosis requires human experts to estimate in a limited time subtle changes in the shape of the optic disc from retinal fundus images. Deep learning methods have been satisfactory in classifying and segmenting diseases in retinal fundus images, assisting in analyzing the increasing amount of images. Model training requires extensive annotations to achieve successful generalization, which can be highly problematic given the costly expert annotations. This work aims at designing and training a novel multi-task deep learning model that leverages the similarities of related eye-fundus tasks and measurements used in glaucoma diagnosis. The model simultaneously learns different segmentation and classification tasks, thus benefiting from their similarity. The evaluation of the method in a retinal fundus glaucoma challenge dataset, including 1200 retinal fundus images from different cameras and medical centers, obtained a $96.76 \pm 0.96$ AUC performance compared to an $93.56 \pm 1.48$ obtained by the same backbone network trained to detect glaucoma. Our approach outperforms other multi-task learning models, and its performance pairs with trained experts using $~\sim 3.5$ times fewer parameters than training each task separately. The data and the code for reproducing our results are publicly available.

## Introduction

Glaucoma is one of the leading causes of irreversible but preventable blindness in working-age populations^1^, which relates to an abnormal fluid balance in the eye that causes an increase in internal ocular pressure. The increased pressure gradually damages the eye optic nerve. If not diagnosed, these induced damages may lead to permanent vision loss. In 2020, it affected approximately 11.2 million people^2,3^.

While an early diagnosis is critical to prevent irreversible damages, patients affected by glaucoma usually do not present symptoms in the early stages of the disease. It is thus essential to develop inexpensive detection methods to massively and systematically control patients before the symptoms appear. One way to achieve this is by performing a visual examination of the posterior pole or retinal fundus image. Specialized cameras obtain the color fundus images in a short image acquisition time. The analysis of the fundus images is performed by ophthalmologists, where the most discriminant symptom for detecting glaucoma on fundus images is the presence of a “cupping,” which is the retraction of the optic disc (OD) on the optic cup (OC). This cupping causes an increase in the vertical Cup-to-Disc ratio (vCDR), which is the height ratio between the OC and OD. Establishing an accurate diagnosis from these images is particularly difficult and prone to error in the accurate estimation of vCDR.

Deep convolutional networks have shown to be beneficial in medical imaging and in tasks of disease classification in eye fundus^4–7^, learning relevant features and patterns directly from images. Over the last years, glaucoma detection using deep learning models reached a remarkable performance at the pair with residents in ophthalmology ^3,8–11^, thus representing a viable alternative to support current visual assessment. However, automating glaucoma diagnosis suffers from lack of data. Existing annotated datasets contain a few hundred samples, while deep learning models require extensive databases to guarantee a good generalization. Moreover, these models include millions of trainable parameters, requiring significant computational resources for training and deployment^12,13^.Therefore, it is essential to develop methods that can make the most from the limited resources: computational requirements and the available annotated images, thus operating in a low data size regime while guaranteeing a good generalization.

Multi-task learning (MTL)^14^ is a learning paradigm that aims to improve generalization by using the domain information contained in the training signals of related tasks as an inductive bias. In practice, this is done by training a shared model for all tasks. In deep MTL, the shared model consists in the parameters of a deep network^15^, hence, the resulting model is smaller than having separate networks for each task. Thanks to these features, MTL is a well-suited approach to automated glaucoma detection, where multiple tasks as OC and OD segmentation, and fovea localization are pre-requisite tasks for computer aided diagnosis (CAD) of retinal diseases^16^. The fovea localization task is related to the OD being located from the center of it by about 2-3 times the diameter of the OD^17^. Despite being related tasks, the use of MTL to simultaneously segment the OD and OC, locate the fovea and detect if the image is glaucomatous has not yet been explored, to the best of our knowledge. Instead, current state-of-the-art works treat each task separately through single task models (STL) or propose MTL approaches that do not exploit the full set of available tasks.

Among STL approaches, Cheng et al.^18^ proposed a super-pixel-based segmentation of the OD and OC for glaucoma screening, achieving a performance in terms of area under the curve (AUC) of 0.822. Fu et al.^19^ obtained 0.899 with a U-Net-based deep learning method and a transformation of the image to polar coordinates. Among the authors that have explored MTL techniques, Mojab et al. proposed a multi-task model for glaucoma detection composed of two modules for OD and OC segmentations and glaucoma prediction^20^, obtaining 90.1 of F-score; the authors did not account for the dissimilarity between the distributions of the segmentation and prediction tasks. Chelaramani reported a novel MTL-based teacher ensemble method for knowledge distillation^21^. The proposed method requires a dataset with a variety of different eye pathologies, which may be difficult to obtain in practice.

This work aims to determine if the relation between tasks associated to glaucoma CAD, i.e. OD and OC segmentation, fovea location and glaucoma detection, can be exploited within an MTL framework to improve model generalization and accuracy for glaucoma detection in a low sample size, low computational resources regime. To this end, a deep MTL model is trained to leverage the similarities of the segmentation of the OD and OC tasks, together with localization of the fovea to detect the presence of glaucoma in retinal fundus images. The proposed MTL approach uses a U-Net encoder-decoder convolutional network as a backbone architecture and adapts it to handle the four tasks using independent optimizers (IO) that can simultaneously learn the segmentation and classification tasks. We denote it MTL-IO. We evaluate our method using the Retinal Fundus Glaucoma Challenge (REFUGE) dataset, including 1200 retinal fundus images (400 for training, 400 for validation, 400 for testing) from different cameras and medical centers, achieving better AUC performance than the same network trained for the single task of detecting glaucoma ($92.91 \pm 0.69$ vs $90.09 \pm 2.70$). Our approach pairs with trained experts^22,23^ and uses approximately 3.5 times fewer parameters than training each task separately.

## Results

This section presents the experimental results obtained on the REFUGE challenge dataset, comparing the proposed MTL-IO framework in different setups and against different baselines.

### Multi-task learning model with independent optimizers

We compared our proposed MTL-IO approach to the respective single task model (STL) for each of the tasks and with two state-of-the-art multitask models: GradNorm^24^ and PCGrad^25^. GradNorm^24^ adaptively balances the losses by gradient normalization, whereas PCGrad is based on estimating the right signs in the independent task gradients to avoid local minima. To gain understanding of the individual contribution of the IO optimization scheme, we also compare our approach to one using the same pipeline, optimized with a standard optimization scheme^14^, which we denote Vanilla MTL. All models were trained five times following a 5-fold cross-validation.

Table 1 shows the classification and segmentation results for each model. Performance is measured in terms of the area under the curve (AUC) for the classification tasks, the Dice score (DSC) for the segmentation tasks, and the L2-distance (Fovea Error) for the localization task. The standard deviation is reported for every performance measure. Model size, in terms of number of parameters (#P), and an iteration time (time), which represents the seconds required for a forward and backward pass in the framework, are also reported.

**Table 1:** Results for the test set in the REFUGE dataset over the four tasks using 5-fold cross-validation.

Models			Tasks			
			Glaucoma	OD	OC	Fovea
Model	#P $\times 1\mathrm {e}{6}$	time	AUC $(\uparrow )$	DSC $(\uparrow )$		Fovea Error $(\downarrow )$
STL	61.2	0.686	$93.56 \pm 1.48$	$\mathbf {95.45 \pm 0.20}$	$86.96 \pm 0.56$	$\mathbf {2.33 \pm 0.33}$
MTL-IO (ours)	${\textbf {17.2}}$	0.557	$\mathbf {96.76 \pm 0.96}$	$95.24 \pm 0.11$	$\mathbf {87.45 \pm 0.90}$	$2.94 \pm 0.24$
Vanilla MTL	${\textbf {17.2}}$	$\mathbf {0.251}$	$94.78 \pm 0.61$	$94.47 \pm 0.25$	$86.45 \pm 0.46$	$5.56 \pm 0.44$
GradNorm	${\textbf {17.2}}$	0.260	$93.47 \pm 2.00$	$94.23 \pm 0.45$	$86.23 \pm 0.65$	$5.56 \pm 0.64$
PCGrad	${\textbf {17.2}}$	0.556	$91.51 \pm 2.44$	$94.76 \pm 0.12$	$87.09 \pm 0.16$	$7.51 \pm 1.37$

Best values are in bold.

MTL-IO outperforms all other methods in glaucoma detection and OC segmentation, while it ranks second in the OD segmentation and the fovea localization task. In terms of model size, all MTL models use approximately $17.2\mathrm {e}{6}$ parameters, making them $~\sim 3.5$ significantly lighter than the STL baseline, which uses $61.2\mathrm {e}{6}$ parameters. We estimate the parameters of STL as the sum of parameters of each single-task learner. In terms of computational time, the cumulative iteration time for STL is $~\sim 1.2$ times slower than MTL-IO. MTL-IO’s training iteration time is comparable to PCGrad, but much slower than GradNorm and Vanilla MTL. This difference is explained by the use of the independent optimizers that incur in a computational overhead, which is compensated by the improved performance.

### Multi-task learning model with independent optimizers and transfer learning

Transfer Learning is a widely adopted method to bias a model with prior knowledge on an input domain and lead it to better generalization on new data. In practice, Imagenet^26^ pre-trained models have proven to be profitable on a large majority of vision tasks. In medical imaging, although the input domain is different from the Imagenet domain (natural images), the benefits are still noticeable^27^, and particularly appreciated to compensate for the usual lack of training data. Its combination with Multi-Task Learning strategies studied here is thus relevant. As Imagenet only involves image classification, there exists no Imagenet pre-trained model for semantic segmentation. However, it is possible to use a pre-trained VGG-16^28^ network for the encoding part of the U-Net in the pipeline, while the decoder is initialized from scratch.

Table 2 presents the results of the different models using a pre-trained encoder. As a reference, we have included a state-of-the-art model proposed for optic disc and cup segmentation^29^, specifically designed to use transfer learning in its pipeline. We denote it Res34-Unet, as it uses a modified U-Net structure, based on a ResNet-34^30^ architecture. To follow their guidelines^29^, we used an encoder pre-trained on the Messidor 2 dataset^31,32^. We observe that MTL-IO improves its performance with the AUC reporting $97.03 \pm 0.59$ in comparison with $96.76 \pm 0.96$ of the MTL-IO strategy with weights trained from scratch. Interestingly, MTL-IO shows a slight drop in performance for OD segmentation. The drop, however, is not significant and can be considered within the model’s variability. The improved performance across tasks is observed for all the other models (Vanilla MTL, GradNorm and PCGrad).

**Table 2:** Results for the test set in the REFUGE dataset over the four different tasks using 5-fold cross-validation.

Model	Tasks			
	Glaucoma	OD	OC	Fovea
	AUC $(\uparrow )$	DSC $(\uparrow )$		Fovea Error $(\downarrow )$
STL	$95.63 \pm 0.57$	$\mathbf {95.68 \pm 0.08}$	$87.45 \pm 0.37$	$\mathbf {2.19 \pm 0.04}$
MTL-IO (ours)	$\mathbf {97.03 \pm 0.59}$	$95.13 \pm 0.34$	$\mathbf {87.55 \pm 0.71}$	$2.34 \pm 0.37$
Vanilla MTL	$96.64 \pm 0.83$	$95.20 \pm 0.24$	$87.53 \pm 0.47$	$3.05 \pm 0.37$
Res34-Unet	−	$93.86 \pm 3.70$	$85.40 \pm 7.46$	−
GradNorm	$95.04 \pm 0.95$	$94.39 \pm 0.31$	$86.38 \pm 0.77$	$5.42 \pm 1.30$
PCGrad	$95.98 \pm 0.58$	$95.32 \pm 0.27$	$87.33 \pm 0.60$	$3.16 \pm 0.30$

The results in this table correspond to the models trained with transfer learning. Res34-Unet contains $25.5 \times {10}^{6}$ parameters. Given the multiple stages of this method, we roughly estimate an iteration time of 0.375s consisting of the training phases through deep networks and excluding any pre/post-processing stages.

Best values are in bold.

### Ablation study: MTL-IO versus single-task learners

**Figure 1:**
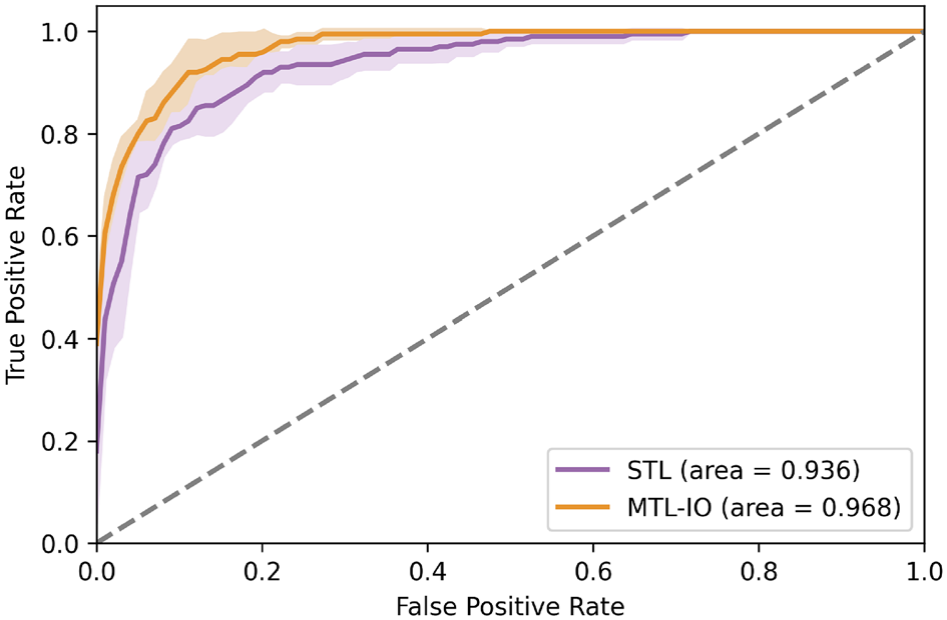
Receiver operating characteristic (ROC) curve for the glaucoma detection task for the single task learning model (STL) and our multi-task learning (MTL-IO) approach.

We investigated in further detail the differences between the proposed multi-task approach and the more standard single-task learner strategy. Figure 1 displays the ROC curves for the glaucoma detection task of STL and MTL-IO. It suggests that the multitask classifier benefits from the related tasks to achieve better performance than the single task of glaucoma detection on all operating points (AUC = 0.968 vs. 0.936).

**Figure 2:**
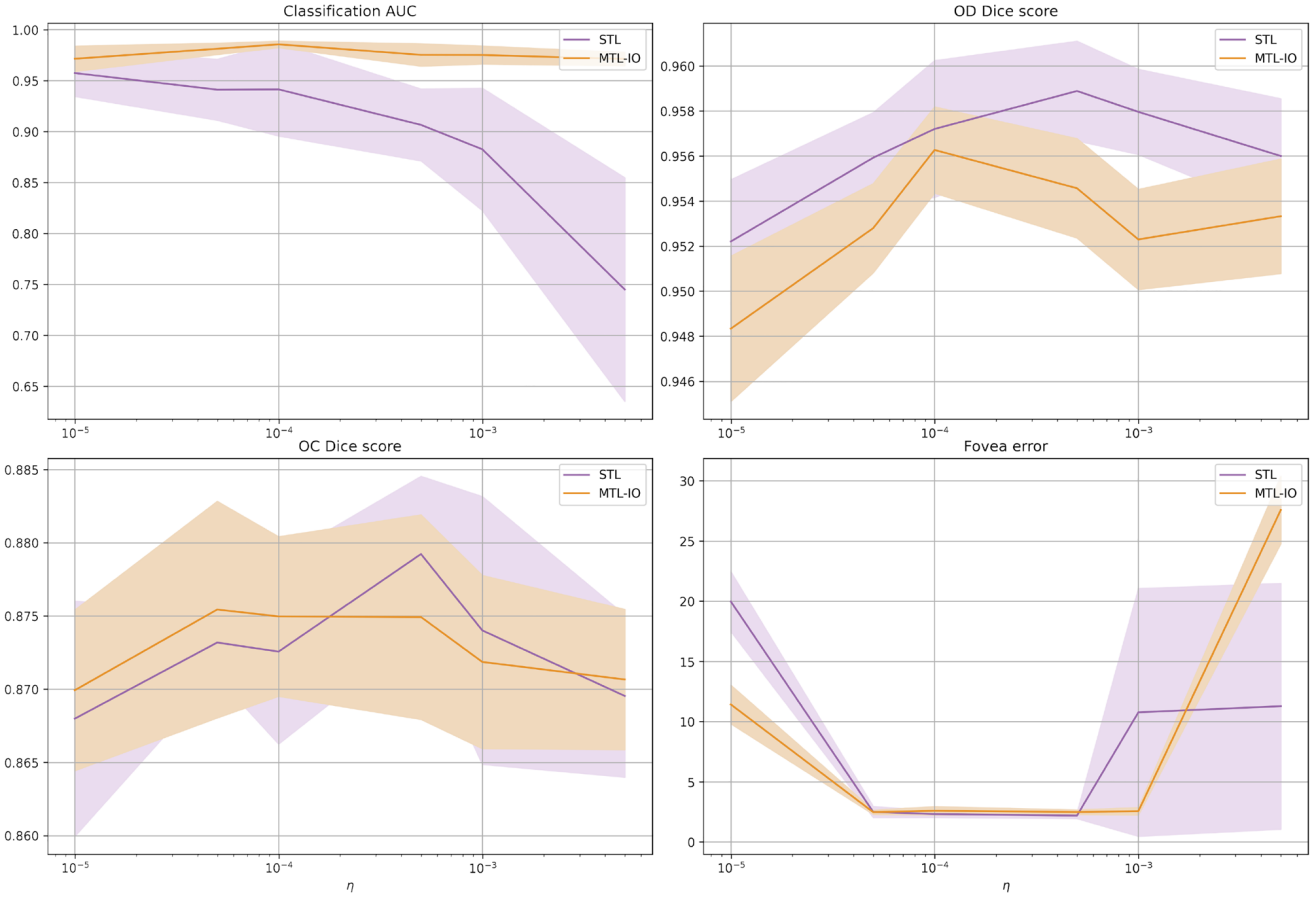
Performance versus learning rate. Per task performance as a function of the learning rate ($\eta$) for the single task learning model (STL) and our multi-task learning (MTL-IO) approach. Standard deviation in shaded colour.

**Figure 3:**
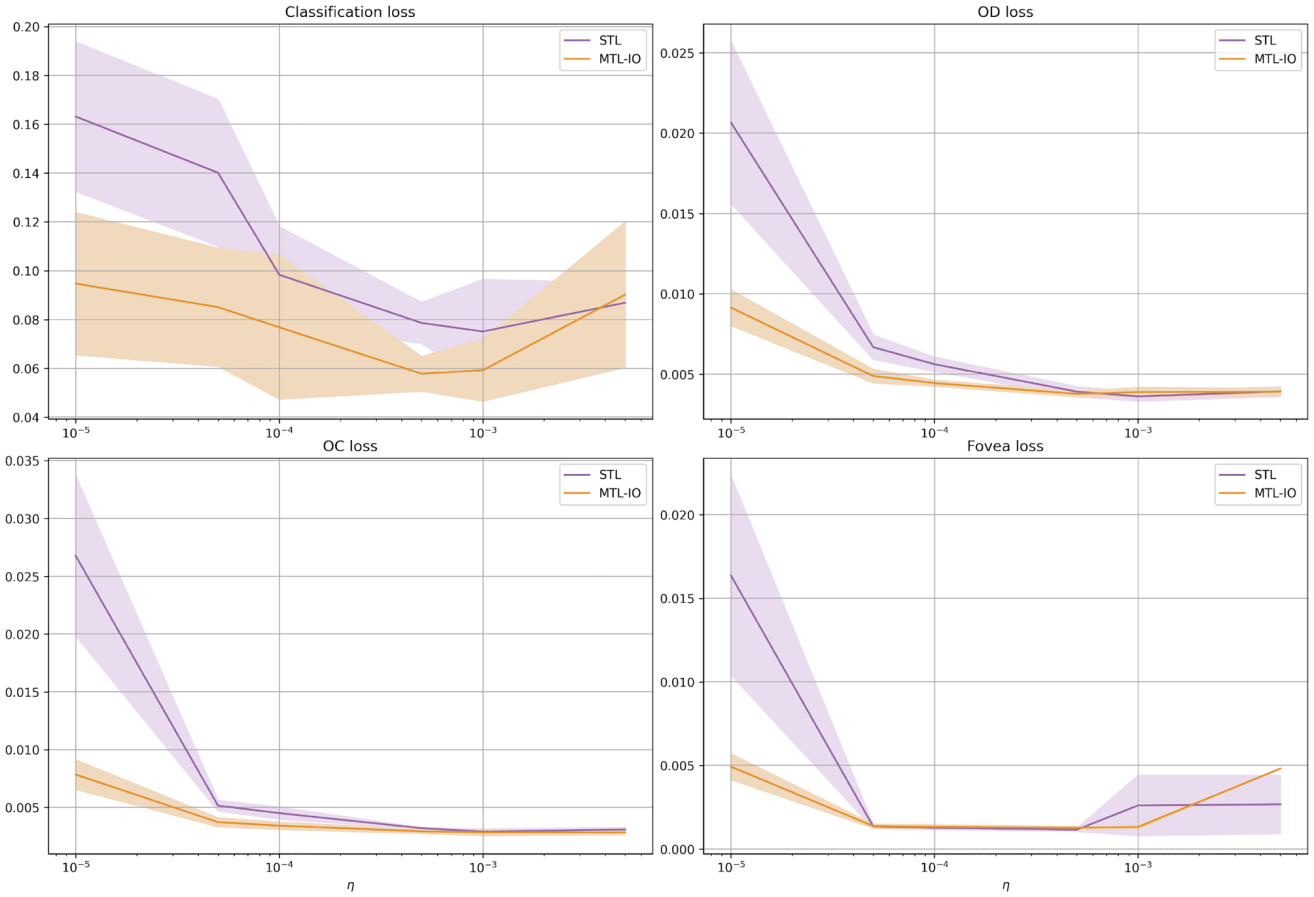
Loss versus learning rate. Final loss values as a function of the learning rate ($\eta$) for the single task learning model (STL) and our multi-task learning (MTL-IO) approach. MTL-IO tends to have lower loss values, suggesting the benefit of learning also from other tasks. Standard deviation in shaded colour.

We also analyzed the sensitivity of MTL-IO to different learning rates at training. Figures 2 and 3 respectively show each task best metric score and minimum loss values, for each of the explored learning rates on the validation set. MTL-IO obtains better results over the different learning rates on the glaucoma detection, while the STL model then performs better on the segmentation tasks, and marginally better on the fovea localization task. However, one can notice that it suffers a more important performance drop on the OC segmentation task when evaluating on the test set (see Table 1), suggesting less overfitting for MTL-IO.

**Figure 4:**
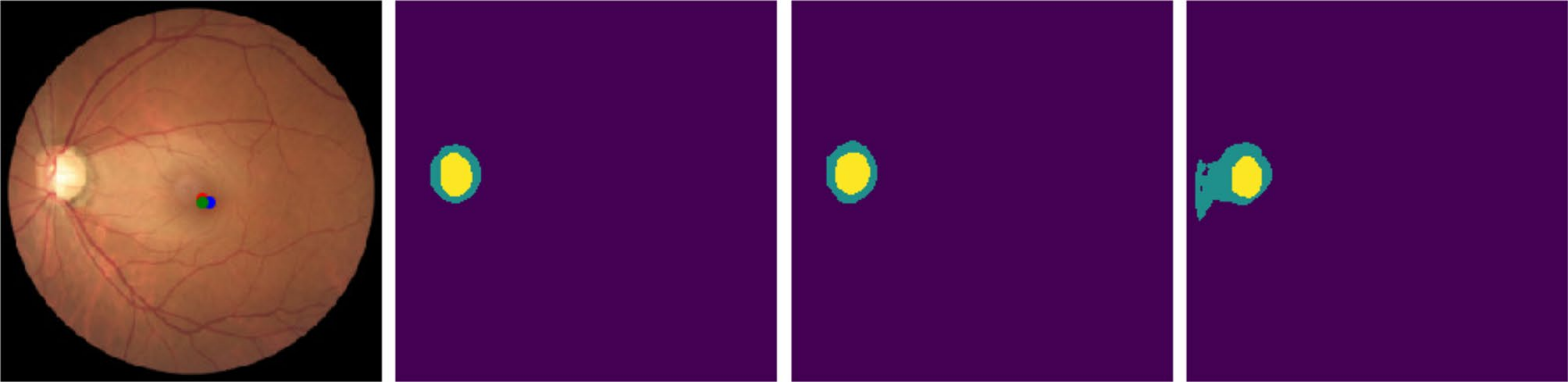
Glaucomatous image from the test set (first image), where the three dots represent the fovea location’s ground truth (red), the MTL-IO prediction (blue) and the STL prediction (green), followed by OD (green) and OC (yellow) ground truth (second), MTL-IO (third) and STL (fourth) segmentation masks.

Figure 4 shows an example of the segmentation of a Glaucomatous eye. The proposed MTL strategy provides a better segmentation in this challenging case, with a distinctive light dome in the middle of the eye, probably due to poor capture conditions. It is a glaucomatous case, although the vCDR does not suggest it.

Finally, and to foster reproducibility, we assessed the performance of both approaches using the official splits proposed by the REFUGE Challenge , i.e. no cross-validation. Instead, the models were trained three times to account for the variation in weights initialization. Tables 3 and 4 summarize the obtained results with and without the use of transfer learning. We observe a drop in the performance for both STL and MTL-IO, which is explained by the distribution shift observed between the challenge’s train and test splits caused by images coming from different imaging devices. In the cross-validation setup, this shift is compensated by the shuffling of the training and validation sets, leading to better results. Despite the drop in performance, MTL-IO remains to be the best performing in terms of AUC.

**Table 3:** Results for the test set in the REFUGE dataset over the four tasks using the challenge’s official splits for training, validation and testing.

Model	Tasks			
	Glaucoma	OD	OC	Fovea
	AUC $(\uparrow )$	DSC $(\uparrow )$		Fovea Error $(\downarrow )$
STL	$87.37 \pm 1.51$	$\mathbf {93.87 \pm 0.74}$	$80.62 \pm 1.25$	$5.96 \pm 0.17$
MTL-IO (ours)	$\mathbf {92.61 \pm 0.38}$	$91.61 \pm 0.64$	$\mathbf {81.21 \pm 0.99}$	$\mathbf {5.71 \pm 0.24}$

Best values are in bold.

**Table 4:** Results for the test set in the REFUGE dataset over the four different tasks with transfer learning using the challenge’s official splits for training, validation and testing.

Model	Tasks			
	Glaucoma	OD	OC	Fovea
	AUC $(\uparrow )$	DSC $(\uparrow )$		Fovea Error $(\downarrow )$
STL	$94.30 \pm 1.68$	$\mathbf {95.29 \pm 0.01}$	$\mathbf {85.86 \pm 0.21}$	$5.42 \pm 0.06$
MTL-IO	$\mathbf {96.15 \pm 0.14}$	$94.24 \pm 0.38$	$83.95 \pm 0.90$	$\mathbf {5.22 \pm 0.18}$

Best values are in bold.

## Discussion

MTL-IO improves generalization by using a unique neural network to learn all tasks jointly. It outperforms all baselines on two of the four proposed tasks , and ranks second behind the STL baseline on the two other tasks while being computationally lighter. Most remarkably, MTL-IO consistently outperforms all baselines on the glaucoma detection task by a large margin.

When comparing the performance of multi-task and single-task models, it is interesting that the other state of the art MTL methods GradNorm^24^ and PCGrad^25^ perform worse than the single task baselines on every task, highlighting a task interference issue. Instead, when using the proposed MTL-IO optimization scheme, the multi-task network can significantly reduce task interference and often improve performances compared to the single-task baselines.

In addition to the improved performance, MTL-IO has the advantage that it uses a unique convolutional network for all tasks. This means that it achieves a good performance while being more lightweight than single-task learners: STL is $\sim 3.5$ times larger in terms of parameters and $\sim 1.2$ times slower than MTL-IO. This is an important feature for real-world use, where resources are often constrained. Our experiments combining transfer learning suggested that the gains achieved by MTL-IO, both in terms of generalization performance and computational efficiency hold in smaller proportions. Although STL observes larger improvements, the MTL-IO remains the best performing at glaucoma detection, which is the main task. As such, it is possible to say that the two strategies, MTL and transfer learning, can be efficiently combined in real-world contexts to create better generalization performance on problems involving multiple tasks.

Despite the above-mentioned advantages, a disadvantage of MTL strategies relates to the extra effort that may be required from a user/expert to put them in place. While an STL strategy requires simple binary labels for training (i.e. presence or absence of glaucoma), MTL techniques also need pixel-wise annotations of the objects to segment and the location of the fovea. All of these annotation tasks are more time consuming and costly. In such setup, it is therefore necessary to assess what is the most critical criterion to optimize. If access to experts for image annotation is difficult, an STL classifier should be used. Instead, if lack of data and limited resources are an important constraint, MTL techniques should be favored.

## Materials and methods

### Materials: REFUGE challenge dataset

In 2018 the *Retinal Fundus Glaucoma Challenge* (REFUGE) was launched as a satellite event at the 2018 MICCAI conference. For this event, 1200 retinal fundus images (400 for training, 400 for validation, 400 for testing) from different cameras and medical centers have been collected and annotated by human experts. Annotations were provided for four different tasks: glaucoma diagnosis, optic disc segmentation, optic cup segmentation, and fovea localization. For the diagnosis task, the ground truth is provided as binary labels, attesting to the presence of glaucoma. In the segmentation tasks, the regions defined by the OD (optic nerve head) and the OC (the white elliptic region located inside the optic disc) are provided as binary segmentations. In the fovea localization case, the ground truth is given as the fovea’s (*x*, *y*) pixel location. All the methods developed and experiments were carried out in accordance with the relevant guidelines and regulations associated to this publicly available dataset.

### Methods

In the following we describe the overall MTL deep learning architecture adopted, the loss functions used for each task and, finally, the independent optimizer (IO) strategy adopted.

#### Multitask deep learning architecture

**Figure 5:**
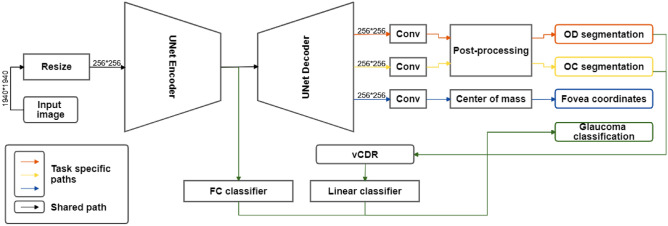
Multi-task learning framework for glaucoma detection, OD and OC segmentation, and fovea localization. The framework uses a U-net as its backbone architecture.

We use a U-Net^33^, an encoder-decoder convolutional network, with a VGG-16^34^ structure and added skip connections between equivalent depths of encoder and decoder, which allow the decoder to recover fine-grained details through the multiple upscalings. This network is well known for solving efficiently biomedical segmentation tasks^35^. Although many variants of the U-Net architecture have been refined for different applications^36^, we choose to use its primary version using a VGG16 architecture, as it is the most widely used, and constitutes a default choice for most applications^36–39^. Our MTL approach uses this architecture for two segmentation tasks (OD and OC), one regression task (fovea coordinates) and one classification task (glaucoma diagnosis). The design of the MTL architecture is shown in Fig. 5, and detailed in the following.

##### Optic disc and cup segmentation tasks

 The OD and OC segmentation masks are obtained through the convolutional layer after the shared decoder for each task. Similar to existing works^9^, the segmentations of OD and OC are refined through a post-processing step that keeps the main connected component in the prediction map to remove possible prediction noise around these elliptic regions.

##### Fovea localization

**Figure 6:**
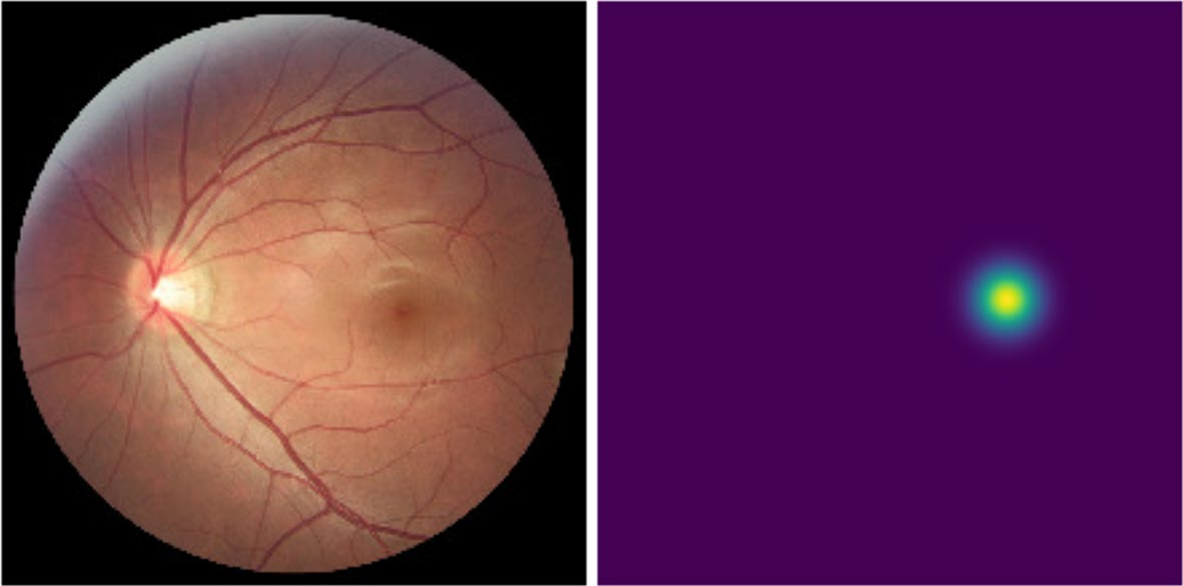
Example of a retinal fundus image (left and the correspondent saliency map centered on the fovea coordinates (right).

 The fovea localization task is addressed as a segmentation task: from the ground truth coordinates of the fovea, a map is created, the center of such map represents the localization of the fovea. The map is a multivariate normal distribution centered in the coordinates (equal variances and null covariances). An example is shown in Fig. 6 (right). The network is trained to fit the maps with a task-respective convolutional layer on the shared decoder. The fovea coordinates are then predicted as the center of mass of the predicted saliency map. In this case, no refinement or postprocessing is performed since it may shift the center of mass.

##### Glaucoma detection task

 The glaucoma detection task (classification) consists of two steps:

1. A prediction is obtained from a fully connected layer, branched after the U-Net encoder (FC classifier).
2. Similarly to some previous works^9^, a second prediction is obtained from a logistic regression classifier (Linear classifier), taking as input the vertical Cup-to-Disc Ratio (vCDR) obtained from the OD and OC segmentation tasks. The vCDR is computed as:
  $$\begin{aligned} vCDR = \frac{OC_{height}}{OD_{height}} \end{aligned}$$
  with $OC_{height}$ and $OD_{height}$ the heights of the OC and OD, obtained from the segmentation branches.

The outputs before the binary outcome of each classifier are averaged. The final classification is obtained by using a threshold of 0.5 over this average.

#### Loss functions

Here, we present the loss functions used for the optimization of the different tasks.

##### OD and OC segmentation

 The OD and OC segmentation tasks both use a binary cross-entropy loss (BCE), averaged over every pixel *i* of the segmentation maps:

$$\begin{aligned} \mathscr{L}_{BCE}(p, y) = -\frac{1}{N_{pix}} \sum _{i=1}^{N_{pix}} y_i \log (p_i) + (1-y_i) \log (1-p_i) \end{aligned}$$

with *p*, *y* and $N_{pix}$ respectively the prediction, ground-truth and number of pixels.

##### Fovea localization

 For the fovea localization task, the network is trained to fit the pre-processed saliency maps with a *L*1-loss, since the map values are not binarized:

$$\begin{aligned} \mathscr{L}_{L1}(p, y) = \sum _i |y_i - p_i| \end{aligned}$$

Afterwards, the predicted fovea location is computed as the center of mass of the predicted saliency map.

##### Glaucoma classification

 For the glaucoma classification task, a focal loss^40^ is used to better handle the unbalance between positive and negative samples (only $10\%$ of positives):

$$\begin{aligned} \mathscr{L}_{Focal}(p, y) = (1-p_t)^\gamma log(p_t) \end{aligned}$$

with

$$\begin{aligned} p_t = {\left\{ \begin{array}{ll} &{} p \quad \text {if} \quad y=1 \\ &{} (1-p) \quad \text {otherwise} \end{array}\right. } \end{aligned}$$

Concretely, this loss multiplies the usual binary cross-entropy term with a classification uncertainty term ($1-p_t$) to give more importance to uncertain classifications, i.e., those of low populated classes. We set the hyperparameter $\gamma$ to 2 in our experiments.

#### MTL independent optimizer optimization strategy

In the following, we present the IO optimization strategy used in this work. It relies on the alternative optimization scheme, alternating independent gradient descent steps on the different task-specific objective functions, as proposed by Pascal et al.^41^. We detail then main steps leading to this optimization scheme, and refer the interested reader to Pascal et al.^41^ for more details.

The standard MTL optimization setup with an aggregated loss^14^ can be expressed as:

$$\begin{aligned} \mathscr{L}(w_t,\xi _t)= \sum _{k=1}^N c^{(k)} \cdot \mathscr{L} ^{(k)} (w_{t}, \xi _{t}) \end{aligned}$$

where $\mathcal {L} ^{(k)}$ is the loss function associated to $k^{th}$ out of *N* tasks, $w_t$ the shared parameters, and $\xi _{t}$ the data sample, at iteration *t*. $c^{(k)}$ are task-specific weighting coefficients, for which we assume uniform weighting, i.e. $c^{(k)} = 1$. If $g^{(k)}$ denotes the derivative of $\mathcal {L} ^{(k)}$ with respect to the shared parameters *w*, the update rule for *w* at step $t + 1$ using stochastic gradient descent is:

1 $$\begin{aligned} w_{t+1} = w_{t} - \eta _t \cdot \sum _{k=1}^N g ^{(k)}(w_{t}, \xi _{t}) \end{aligned}$$

where $\eta _t$ is the learning rate.

Recent works^15,42,43^ propose a variation to the update rule in equation 1, in which alternate independent update steps with respect to the different task-specific loss functions are executed, instead of aggregating all the terms at once. This strategy aims to minimize task interference and, hence improve generalization. The alternate update rule can be expressed as:

2 $$\begin{aligned} w_{t+1}^{(k)} = {\left\{ \begin{array}{ll} w_{t}^{(N)} - \eta _t \cdot g^{(k)} ( w_{t}^{(N)},\xi _t), &{} k=1 \\ w_{t}^{(k-1)} - \eta _t \cdot g^{(k)} ( w_{t}^{(k-1)},\xi _t), &{} \forall k > 1 \\ \end{array}\right. } \end{aligned}$$

In this work, we adopt the approach from Pascal et al.^41^. It uses a modified alternate update rule (eq. 2) that allows to use individual optimizers (IO) in the form of individual exponential moving averages for each task, to prevent state-of-the-art optimizers (e.g. Adam) from accumulating and mixing previous gradient descent directions of all the different tasks. The modified update rule can be expressed as:

3 $$\begin{aligned} w_{t+1}^{(k)} = {\left\{ \begin{array}{ll} w_{t}^{(N)} - \eta _t \cdot \hat{m}^{(k)} \left( g^{(k)} ( w_{t}^{(N)},\xi _t) \right) , &{} k=1 \\ w_{t}^{(k-1)} - \eta _t \cdot \hat{m}^{(k)}\left( g^{(k)} ( w_{t}^{(k-1)},\xi _t) \right) , &{} \forall k > 1 \\ \end{array}\right. } \end{aligned}$$

where $\hat{m}^{(k)}$ is a task-specific exponential moving average mechanism. Here, the memory term introduced by $m^{(k)}$ only involves previous updates of task *k*. Such formulation is equivalent to using one independent optimizer per task, and is therefore denoted as MTL-IO. In this paper, we use MTL-IO to denote the complete pipeline.

### Implementation details

All methods were implemented in Pytorch 1.2, and ran on NVIDIA Titan XP graphic cards. Kaming uniform initialization^44^ was used for all the baselines, except for network parts initialized with transfer learning. For the 5-fold cross-validation, the validation splits were defined on the merged and shuffled train and validation official splits, while the test split was kept unchanged.

## Data Availability

The data used to train our models and run experiments is available, upon registration from the REFUGE Challenge (https://refuge.grand-challenge.org/Home2020/). All code to reproduce the results of this article is available in a GitHub repository (https://github.com/robustml-eurecom/glaucoma_mtl). The code can be anonymously downloaded from the following link: https://github.com/robustml-eurecom/glaucoma_mtl/archive/refs/heads/main.zip.
